# Developing a single-stage continuous process strategy for vitamin B_12_ production with *Propionibacterium freudenreichii*

**DOI:** 10.1186/s12934-023-02029-x

**Published:** 2023-02-09

**Authors:** Álvaro Calvillo, Teresa Pellicer, Marc Carnicer, Antoni Planas

**Affiliations:** 1 Laboratory of Biochemistry, Institut Químic de Sarrià, University Ramon Llull, 08017 Barcelona, Spain; 2 HealthTech Bio Actives, 08029 Barcelona, Spain

**Keywords:** Cobalamin, *Propionibacterium freudenreichii*, Cyanocobalamin production, Fed-batch culture, Continuous culture

## Abstract

**Background:**

Vitamin B_12_ is a widely used compound in the feed and food, healthcare and medical industries that can only be produced by fermentation because of the complexity of its chemical synthesis. Besides, the use of Generally Recognized as Safe (GRAS) and Qualified Presumption of Safety (QPS) microorganisms, like *Propionibacterium freudenreichii,* especially non-GMO wild-type producers, are becoming an interesting alternative in markets where many final consumers have high health and ecological awareness. In this study, the production of vitamin B_12_ using the *Propionibacterium freudenreichii* NBRC 12391 wild-type strain was characterized and optimized in shake flasks before assessing several scale-up strategies.

**Results:**

Initial results established that: (i) agitation during the early stages of the culture had an inhibitory effect on the volumetric production, (ii) 5,6-dimethylbenzimidazole (DMBI) addition was necessary for vitamin B_12_ production, and (iii) kinetics of vitamin B_12_ accumulation were dependent on the induction time when DMBI was added. When scaling up in a bioreactor, both batch and fed-batch bioprocesses proved unsuitable for obtaining high volumetric productivities mainly due to carbon source limitation and propionic acid inhibition, respectively. To overcome these drawbacks, an anaerobic single-phase continuous bioprocess strategy was developed. This culture strategy was maintained stable during more than 5 residence times in two independent cultures, resulting in 5.7-fold increase in terms of volumetric productivity compared to other scale-up strategies.

**Conclusion:**

Overall, compared to previously reported strategies aimed to reduce propionic acid inhibition, a less complex anaerobic single-phase continuous and more scalable bioprocess was achieved.

**Supplementary Information:**

The online version contains supplementary material available at 10.1186/s12934-023-02029-x.

## Introduction

Vitamin B_12_, also known as cobalamin (Cbl), is a water-soluble molecule essential in many organisms. This molecule was first studied in the 1920s due to its important role in the prevention and treatment of serious diseases like pernicious anemia [1]. Although it was not until 1948 that was isolated and described by two research groups from pharmaceutical companies (Merck, Sharp & Dohme, and Glaxo [2, 3]). Later, this red crystalline compound was found to be, in fact, a family of molecules that shared a similar structure: a tetrapyrrolic corrinic ring with a central cobalt atom coordinated to four nitrogen atoms [4, 5], a similar scaffold to other prosthetic groups such as the heme group of hemoglobin or the electron transport chain group of cytochrome P450. In addition, besides the four N atoms of the pyrrole units, the central Co^+^ ion is linked to two other ligands. The lower ligand in an α-axial conformation is 5,6-dimethylbenzimidazole (also known as DMBI) while the upper ligand is linked to the Co^+^ ion in the β-axial position. The nature of this last chemical group is variable and defines the different physiological and catalytic functions of cobalamins such as methylcobalamin (MetCbl), a cofactor of several methyltransferases [6, 7], and adenosylcobalamin (AdoCbl), a cofactor of mutases like the l-methylmalonyl-CoA mutase found in mammals [8].

All these molecules present a very complex structure and an elaborated biosynthesis with over 30 biotransformation steps [9]. For this reason, although the chemical synthesis of vitamin B_12_ has been established since the 1970s [10, 11] fermentation processes are preferred for cobalamin production. Moreover, the most common commercial form of vitamin B_12_ is not MetCbl or AdoCbl but cyanocobalamin (CNCbl), which is a more stable form of vitamin B_12_ and readily converted into the active coenzyme forms in the body [12]. Nowadays, industrial vitamin B_12_ production with *Pseudomonas denitrificans (P.denitrificans)* strains have been favored due to their faster growth rate and productivity [13, 14], displacing other traditionally employed anaerobic strains such as *Propionibacterium freudenreichii (P. freudenreichii).* However, *P. freudenreichii* still presents properties that make it an interesting candidate for cobalamin production. For example, in contrast to *P. denitrificans*, it is a Generally Recognized As Safe (GRAS) microorganism and has the Qualified Presumption of Safety (QPS) status granted by the EFSA. Moreover, it can grow in a wide range of different and inexpensive carbon and nitrogen sources [15–17] or produce other valuable compounds like propionic acid or trehalose [18].

Vitamin B_12_ supplementation is gaining relevance in recent years with the rise in vegan and vegetarian populations [19]. For this reason, the use of GRAS and QPS producers, like *P. freudenreichii*, especially non-GMO wild-type producers, are becoming an interesting alternative in markets where many final consumers have high health and ecological awareness. In addition, different *P. freudenreichii* strains have been traditionally used for cheese and other food productions facilitating the development of several in-situ fortification strategies without the need for direct supplementation [20, 21].

Traditionally, Cbl production in *P. freudenreichii* has been performed in a two-stage fermentation process: a first stage completely anaerobic aimed at cell growth followed by a microaerophilic production stage, needed for DMBI synthesis and its attachment to the corrinic ring [22, 23]. The main drawback of *P. freudenreichii* Cbl production compared to aerobic strains, besides its slower growth and lower volumetric productions, is the large quantities of propionic acid produced through the Wood-Werkman cycle [24]. To reduce this latter limitation, main industrial *P. freudenreichii* producers have been obtained through random mutagenesis processes where strains with high tolerance to propionic acid were selected [25]. Other approaches, like genetic engineering [26, 27], several strategies to decrease propionic acid concentration, such as expanded bed adsorption bioreactors [28, 29], co-culture with propionic consuming strains [30] and supplementation with different Cbl precursors [23, 31], were tested in the past, although results are often very strain dependent and further characterization and optimization is still needed.

In this study, a bioprocess with a wild-type non-propionic resistant strain of *P. freudenreichii* was optimized for cobalamin production. The selected strain was NBRC 12391, also named IFO 12391, reported as producing strain by Kojima and co-workers [25]. To increase market accessibility, all the improvements performed were done at a bioprocess level, without any genetic modification. Besides, industrial cheap media were preferred such as corn steep liquor (CSL), a byproduct of the mill industry, over more expensive alternatives like yeast extract (YE), even though the combination of both CSL and YE has recently been reported to have a positive effect on cobalamin production [32].

Initially, aeration in early stages of the culture and the addition of different precursors were found to affect the final volumetric production. Later, these results were scaled from shake flasks to a lab-scaled bioreactor. Finally, thanks to the specific cobalamin production kinetics of our strain, a single-phase continuous bioprocess was proposed and developed, in contrast to the usually described two-stage culture [33], which minimized propionic acid inhibition and maximized cobalamin productivity. Compared to previous studies where an increased production was obtained by eliminating the propionic acid from the media with several resin systems [29, 34, 35], a less complex and more scalable bioprocess was achieved.

## Results and discussion

### Culture characterization in shake-flasks

#### Agitation effect during the early culture stage

Agitation is mandatory in any culture system to get a homogeneous system and not promote nutrient gradients or cell sedimentations. Nevertheless, previous studies with *P. freudenreichii* strain have concluded that agitation may have an inhibitory effect on CNCbl production during the production stage [36]. This information may become important in scaling up the bioprocess as bioreactor systems are always agitated so, a potential effect on growth or CNCbl production needs to be properly characterized on each strain.

In preliminary experiments performed with gentle agitated shake flasks, the final CNCbl productions obtained were lower than those described in the literature for wild-type strains [37] with values below 2 mg/L. Because of these results, and even though some amount of agitation will be needed in the eventual scale-up of the process, a culture methodology based on static shake flasks was assessed.

**Fig. 1:**
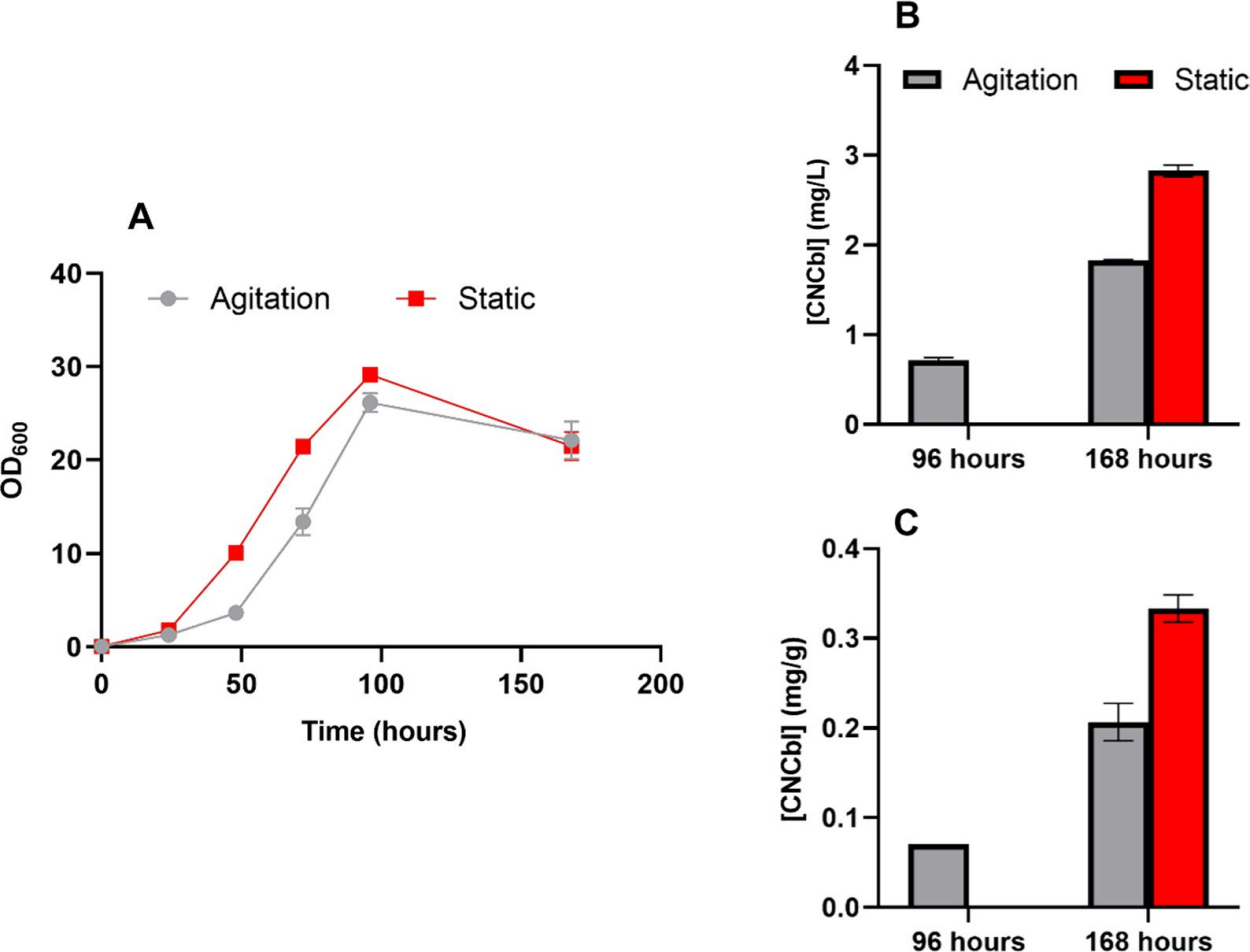
**A** OD_600_ values, **B** volumetric productions (mg CNCbl/L) and **C** specific productions (mg CNCbl/g Biomass) at 96 and 168 h of both conditions, agitated or static. Agitated cultures were kept at 150 rpm during the whole culture while static cultures were kept without any kind of agitation during the first 96 h and gentle agitated at 150 rpm afterwards. In both cases, 100 µM DMBI was added at 96 h to promote Cbl production. Error bars represent the standard deviation of three replicates

Figure 1 shows the results obtained from shake flask cultures which have been gently agitated (150 rpm) or maintained in static conditions. From the OD_600_ profile, a slightly delayed growth was observed on agitated cultures from the beginning, but both conditions reached similar maximum values at 96 h, OD_600_ of 29.1 ± 0.3 and 26.1 ± 1.0 for the static and agitated cultures respectively. These results seem to suggest that agitation may have a slightly negative effect on cell growth, especially during the early stages of the culture.

As previously described elsewhere [13], at 96 h CNCbl production was induced by adding DMBI and promoting a microaerophilic condition by gently agitate both conditions. Unexpectedly, our strain was already producing a significant amount of CNCbl before the induction but only under agitation, 0.71 ± 0.03 mg/L, in contrast to the static condition where CNCbl was under the quantification limit of our method (0.3 mg/L). However, at the end of the culture, the static condition presented significantly higher volumetric and specific production, 2.83 ± 0.06 mg/L and 0.33 ± 0.02 mg CNCbl/g Biomass respectively, indicating that the final production depends on the culture condition before the DMBI addition.

Other *P. freudenreichii* wild-type strains presented similar values compared to our static condition in terms of CNCbl volumetric production. For example, Liu et al. reported productions with their non-optimized media of around 3.81 mg/L [32] and Chamlagain and coworkers reported a production of 5.3 mg/L in whey-based media [23].

Although an agitate system is needed upon scaling, the static condition was selected as the culture condition for further strain characterization in shake flask setups. We assumed that the slight inhibitory effect apparently produced by an early agitation was due to minor oxygenation that may have compromised the anaerobic condition. In a bioreactor set-up, an anaerobic condition can be better ensured, for example, by the constant addition of N_2_ to the vessel, so this effect can be easily avoided.

#### Supplementation of CNCbl precursors

**Fig. 2:**
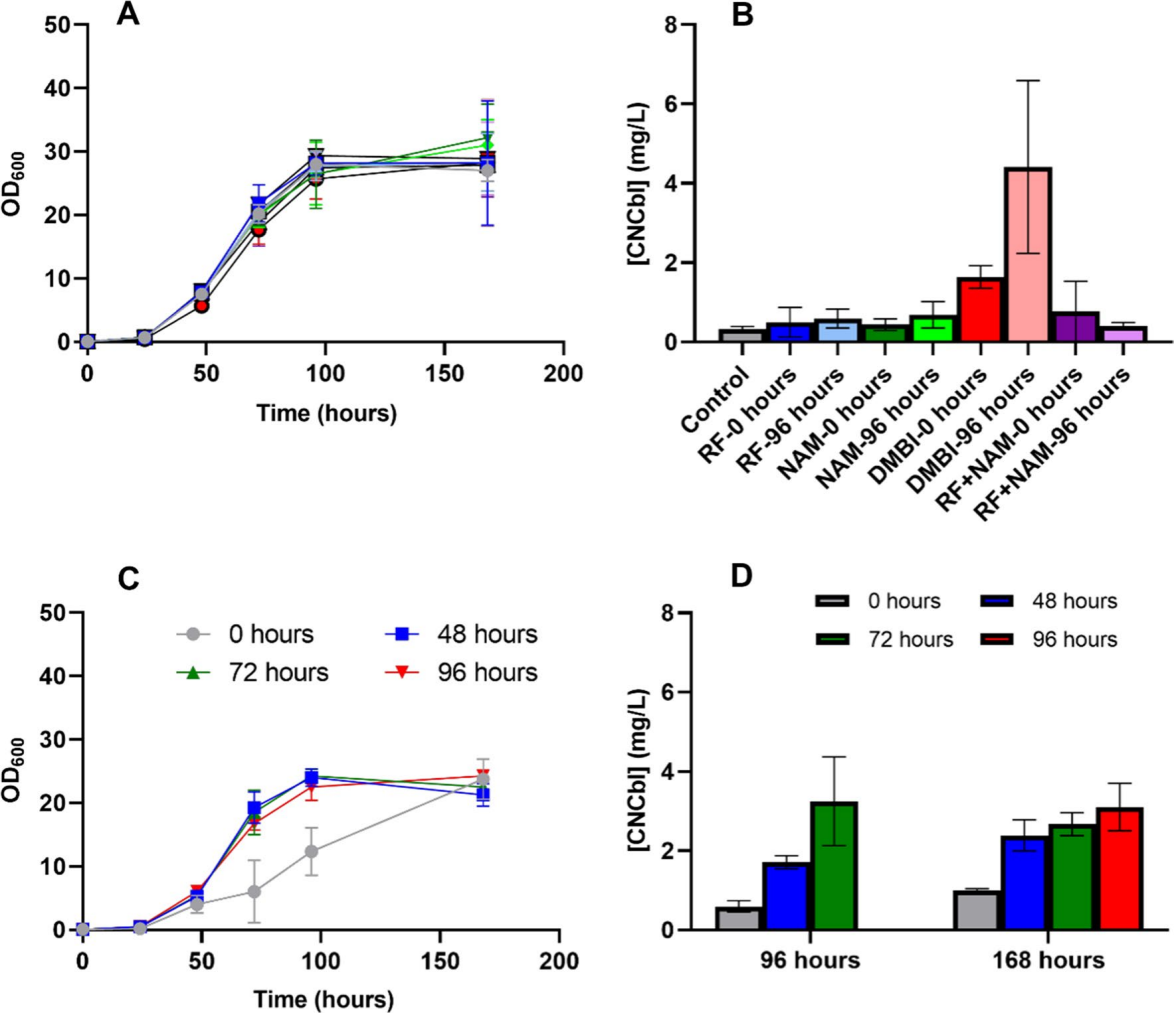
**A** and **B** OD_600_ values and volumetric productions (mg CNCbl/L) at 168 h of NBRC 12,391 cultures where different Cbl precursors were added to the culture at 0 or 96 h. Control (without precursors), Riboflavin (RF), Nicotinamide (NAM), 5,6-dimethylbenzemidazole (DMBI) and a combination of both riboflavin and Nicotinamide (RF + NAM). **C** OD_600_ values and **D** volumetric productions (mg CNCbl/L) at 168 h of NBRC 12391 cultures where DMBI was added at four different times: 0, 48, 72 and 96 h. Error bars represent the standard deviation of three (**A** and **B**) or two (**C** and **D**) replicates

Classical cultures aimed at CNCbl production often use DMBI addition to induce further accumulation in the microaerobic stage, but there are other precursors such as riboflavin (RF) or nicotinamide (NAM) that have been reported to work for other wild-type producing strains [23]. To define the best supplementation approach for our strain (NBRC 12391), 100 µM of RF, 100 µM of NAM or 100 µM of DMBI were added at two different culture stages, at 0 and 96 h (Fig. 2). Moreover, 50 µM of RF plus 50 µM of NAM were combined to test potential synergistic effects of both precursors.

The results showed that, from all conditions tested, only supplementation with DMBI at time 0 seemed to have a slightly negative effect on cell growth during the early stages of the culture (Fig. 2A). Nevertheless, despite this inhibitory effect, the final absorbance achieved was not significantly different from the rest of the tested conditions, with OD_600_ values of around 30 at 168 h.

CNCbl production was assessed only at 168 h, the final point of the culture after 72 h of gently agitation (Fig. 2B). At this time, all conditions tested including the control condition without any addition showed some amount of CNCbl production. However, except for DMBI, the addition of precursors, independently of when they were added, did not significantly differ from the control condition. Thereby, for our strain, only DMBI was able to induce higher CNCbl accumulation and the other precursors were discarded for future experimentation. In addition, and as previously described [38], it was better to add DMBI at the beginning of the microaeration stage, 96 h, and not from the beginning.

To further optimize DMBI addition, four different addition times were tested, 0, 48, 72 and 96 h (Fig. 2C and D). Like previous experiments, DMBI addition at time 0 resulted in a slower growth rate during the early stages of the culture, but the effect was more pronounced in this later study. Nevertheless, the final OD_600_ value was similar to the rest of the conditions tested.

As shown in Fig. 2D, CNCbl production was evaluated at 96 and 168 h with DMBI inductions at 0, 48, 72, and 96 h. As expected, at 96 h all conditions presented significant amounts of CNCbl, being the induced culture at 72 h the best presenting already 3.3 ± 1.2 mg/L of CNCbl just 24 h after the induction. Moreover, the production at 168 h when DMBI was added at 96 h (3.11 ± 0.6 mg/L) was not significantly different from the values obtained at 96 h when DMBI was added at 72 h. These results suggested that, after certain OD_600_ value of around 20 is achieved, the maximum CNCbl volumetric production in NBRC 12391 cultures can be obtained just 24 h after DMBI addition.

### Scale-up. Batch and fed-batch processes

The scale-up strategy began with the development of a classical batch bioprocess in a laboratory-scale bioreactor with 1 L working volume. For the first 96 h, a strict anaerobic condition was set by pumping N_2_ constantly at 0.15 vvm. Although agitation was found to reduce the final volumetric production in shake flasks, we fixed a low agitation of 150 rpm through all the processes to ensure homogenization in the bioreactor and avoid cell sedimentation.

#### Batch with DMBI induction at 96 h

**Fig. 3:**
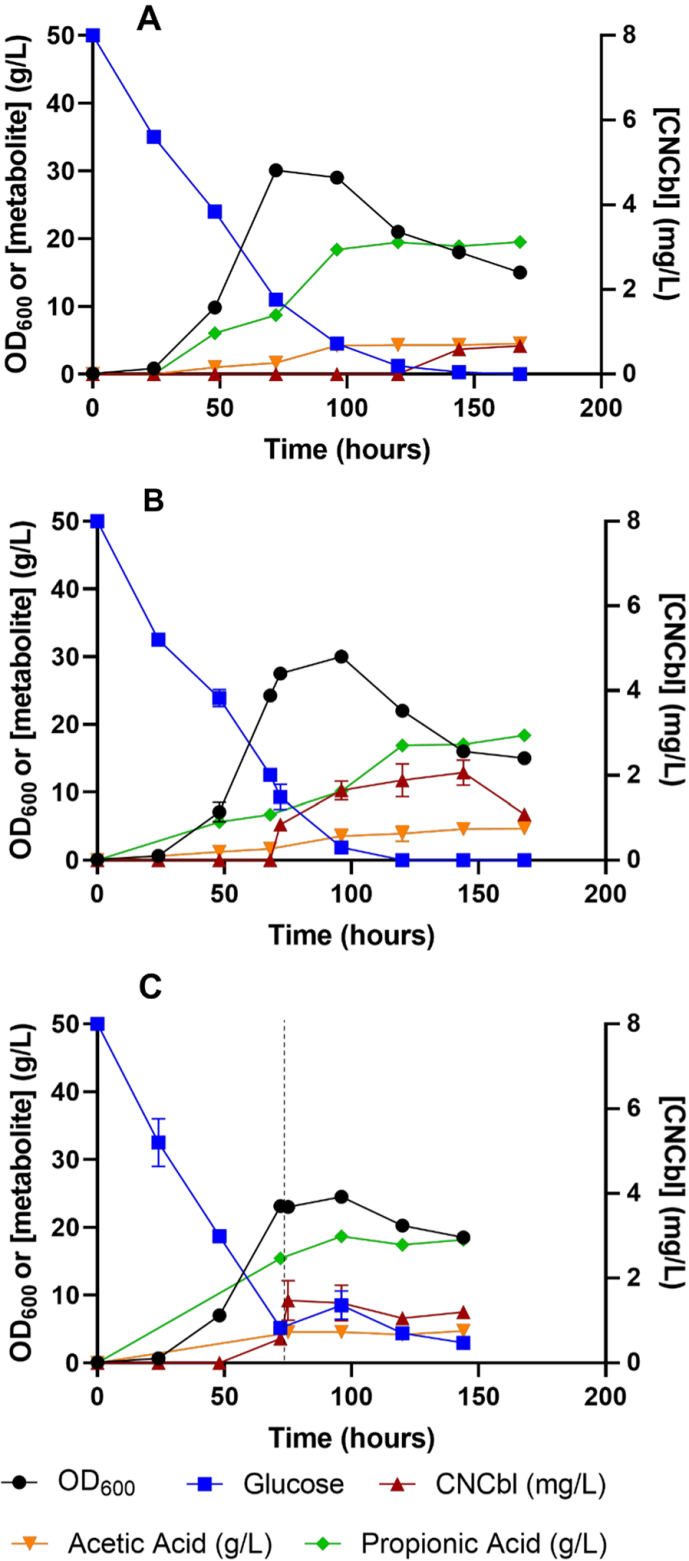
**A** Batch with DMBI addition at 96 h. **B** Batch with DMBI addition at 72 h. **C** Fed-batch with DMBI addition at 72 h. OD_600_ values, glucose (g/L), acetic acid (g/L), propionic acid (g/L) and CNCbl (mg/L) are provided. The vertical dashed line in the fed-batch process represents when feeding was started. Each process was performed by duplicate except the Batch process with DMBI addition at 96 h with only 1 replicate

Initial batch experiments were performed according to previously reported strategies for other producing strains where the growth phase was extended for 96 h. After that time, 100 µM DMBI was added to the culture, enough to produce up to 50 mg/L of vitamin B_12_, and N_2_ addition was stopped to promote microaeration. The growth profile and metabolite productions of the NBRC 12391 strain in a batch culture are represented in Fig. 3A. The obtained data shows that our strain presented faster growth rates in the bioreactor, even though it was agitated, compared to shake flasks studies as the maximum OD_600_ was obtained sooner, at approximately 72 h. These results were expected as pH control was much better in the bioreactor than in shake flasks that were manually controlled daily. Moreover, as it grew faster, when DMBI was added at 96 h, glucose was almost depleted presenting values around 4 g/L. Under the microaeration stage, glucose was completely depleted from the media and OD_600_ values began to decrease, most probably due to some degree of cell lysis. This may be also the reason why, even though CNCbl production began when DMBI was added, the final volumetric production values were significantly lower than the ones obtained in shake flask cultures, 0.63 mg/L. Besides biomass and CNCbl production, acetic and propionic acid accumulations were also followed through the process. As expected, propionic acid was the principal fermentation by-product mainly produced during the anaerobic phase reaching 18.4 g/L at 96 h. This value remained stable in the microaerobic phase (reaching a maximum value of 19.5 g/L at 168 h) most probably because glucose was already almost depleted.

#### Batch with DMBI induction at 72 h

Taking into consideration previous experiments where it was proved that maximum volumetric production values could be obtained with earlier DMBI addition if certain OD_600_ values were achieved, other batch processes were performed with DMBI addition at 72 h, with OD_600_ values of 27.5 and a glucose concentration of 8.5 ± 0.7 g/L. After DMBI addition, CNCbl production began, and maximum volumetric values were 2.05 mg/L ± 0.3 in a 48-h period (Fig. 3B). Interestingly, at 75 h, just 3 h after the DMBI addition we could already quantify 0.85 mg/L ± 0.02 of CNCbl suggesting a fast accumulation of the vitamin. Nevertheless, the volumetric production of 2–4 mg CNCbl/L obtained in static shake flask cultures could not be achieved. On the other hand, comparing the propionic acid production, the early CNCbl induction promoted a slower production rate of this acid at the beginning of the microaerobic phase but it finally reached a similar concentration at 120 h.

#### Fed-batch with DMBI induction at 72 h

Due to the impact of carbon source limitation on both OD_600_ and CNCbl volumetric production, a fed-batch bioprocess with constant glucose addition after 72 h was assessed (Fig. 3C). Despite the increased glucose concentration in the culture, OD_600_ did not reach higher values and, in fact, OD_600_ max (24.5 ± 0.71) was lower than the one obtained in the batch bioprocesses. Besides, maximum volumetric production was also lower (1.48 mg/L ± 0.46). These results suggested that the growth and production of our strain were limited not only by the lack of carbon source but also by other factors such as propionic acid inhibition. The latter effect has been widely reported previously [32] and propionic concentration in the final stages of the cultures was probably too high for our strain, around 18 g/L in the batch processes. Considering this inhibition, a fed-batch strategy without a mechanism for propionic acid elimination/removal (for example, an expanded bed reactor like the one used in [34]) does not seem to be a viable strategy for cobalamin production.

### Development of a single-stage continuous bioprocess in lab-scaled bioreactor for increased CNCbl volumetric productivity.

#### Propionic acid inhibition effect on culture growth and anaerobic production of CNCbl

Considering the results obtained in the fed-batch culture experiments, developing a strategy to decrease the inhibitory effect of high propionic acid concentrations seemed to be mandatory to increase production with the NBRC 12391 strain. Moreover, the two-stage production process with an anaerobic growth phase and a microaerobic production phase severely hinders the viability and increases the complexity of such bioprocess. A single-phase process, with constant anaerobic conditions to ensure cell growth would be preferable. Nevertheless, for this strategy, our strain should be capable of producing CNCbl in anaerobic conditions without a microaerobic production stage. As the majority of the needed O_2_ is employed in DMBI biosynthesis, a process not needed in our bioprocess as DMBI is added in excess as a supplement, made us consider a single-stage continuous strategy. Besides, the fact that maximum production of our strain was achieved just 24 h after DMBI addition (see Fig. 2D) was promising and let us develop a bioprocess with a residence time of less than a day without losing CNCbl production.

**Fig. 4:**
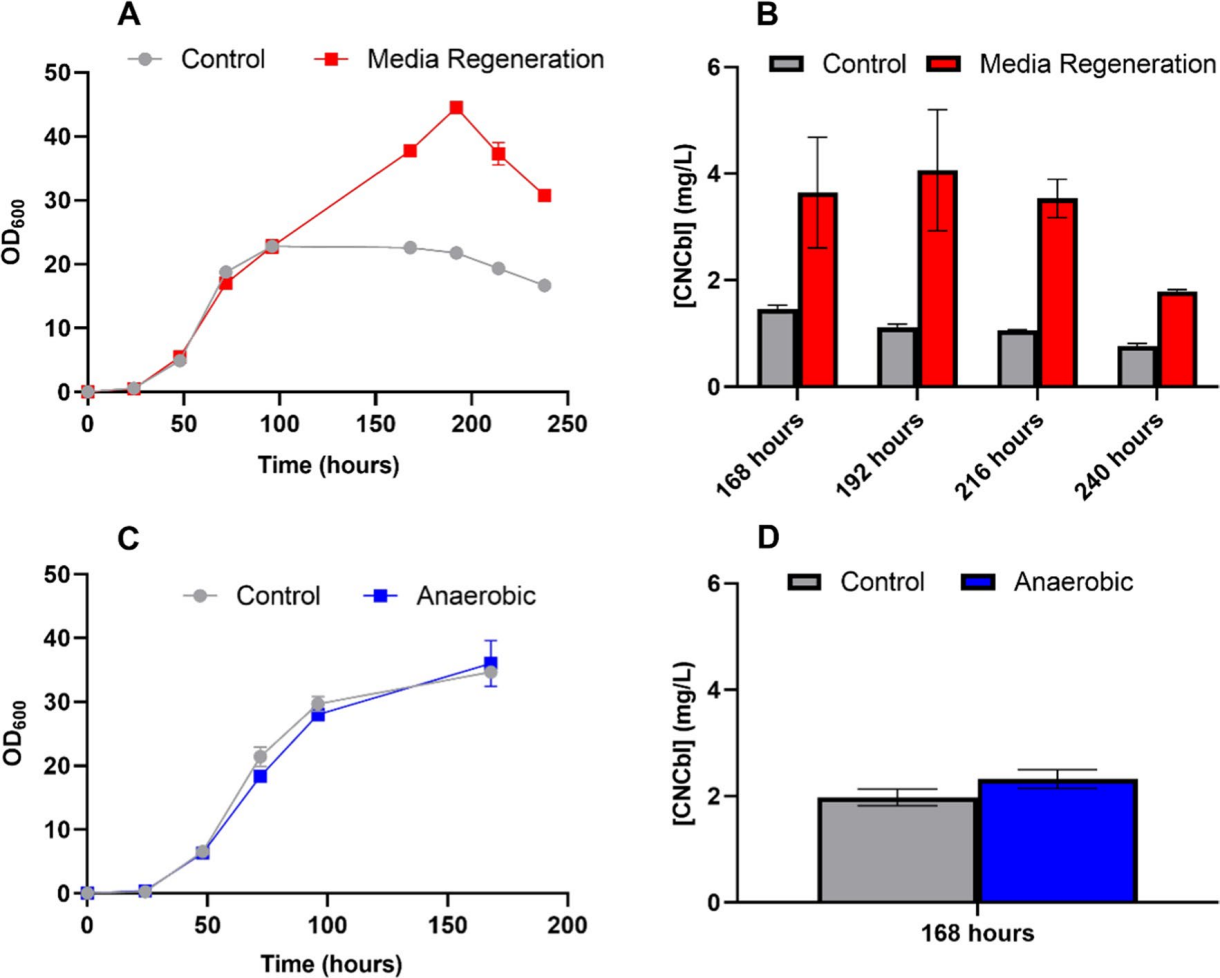
**A** and **B** OD_600_ values and volumetric productions at 168, 192, 216 and 240 h of NBRC 12391 control cultures and cultures where the cells were centrifuged and resuspended in fresh media at 96 h. In both cases, 100 µM DMBI was added at 96 h to promote Cbl production. **C** and **D** OD_600_ values and volumetric productions at 168 h of NBRC 12391 control cultures and cultures that were kept anaerobic through the whole process. In both cases, 100 µM DMBI was added at 96 h to promote Cbl production. Error bars represent the standard deviation of three replicates

Before developing the bioreactor process, several experiments were performed in shake flasks to ensure the viability of the strategy. First, the capacity of the cell culture to continue growing in fresh media was tested. To do so, after the first 72 h of growth, the whole culture was centrifuged, the supernatant discarded, and the cell pellet resuspended in fresh media and supplemented with 100 µM DMBI (Fig. 4A and B). This strategy allowed to reach not only higher OD_600_ values, 44.5 ± 0.7 *vs* 22.75 ± 1.1 in the control culture (cultures performed without media regeneration as described in Section “Culture characterization in shake-flasks”) but also higher CNCbl volumetric production, 4.06 ± 1.14 mg CNCbl/L *vs* 1.42 ± 0.07 mg CNCbl/L. Therefore, as expected, we proved that eliminating propionic acid from the media allowed the culture to continue growing and producing.

As previously mentioned, one of the main challenges for the development of a continuous process for *P. freudenreichii* is the reported necessity of a microaerobic production phase after the strictly anaerobic growth phase. To test if NBRC 12391 was capable of achieving similar CNCbl production in a single anaerobic phase, several cultures were performed in shake flasks kept at static conditions during the whole 168 h process (Fig. 4C and D). As a result, OD_600_ values and CNCbl volumetric and specific productions were not significantly different between the conditions, thereby, allowing a single anaerobic process.

#### Continuous bioprocess in bioreactor

With the objective of reducing the propionic acid inhibition in the vessel at steady state, a continuous culture process was designed with lower carbon and nitrogen source concentrations. More specifically, the initial culture media and the fed media contained 25 g/L glucose and 20 g/L CSL instead of 50 g/L and 40 g/L used in the batch bioprocesses respectively. Thereby, we were expecting amounts of 9 g/L of propionic acid at steady state assuming equivalent yields as in previous Batch processes (See Fig. 3B).

**Fig. 5:**
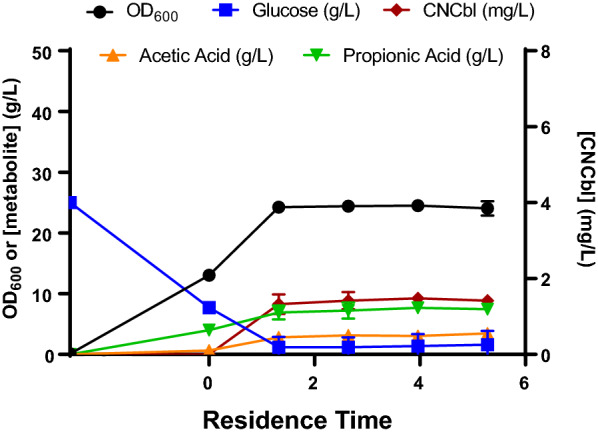
Continuous bioprocess of NBRC 12391. The number of residence times is shown on the X-axis. According to the calculated addition of both fresh media and NaOH solution, each residence time corresponds to 18.2 h. Fresh media containing 100 µM DMBI addition began at residence time 0. OD_600_ values, glucose (g/L), acetic acid (g/L), propionic acid (g/L) and CNCbl (mg/L) are provided. Error bars represent the standard deviation of two replicates

After batch phases of 48 h (Fig. 5), before complete glucose depletion, OD_600_ of 13.05 ± 0.07 were reached, 100 µM DMBI was added and the continuous bioprocesses were begun with the constant additions of fresh media (containing 100 µM DMBI) at a flow rate of 50 mL/h. The total volume addition rate was 55 mL/h due to the constant addition of 2 M NaOH (5 mL/h) to control pH. These values imply a dilution rate of 0.055 h^−1^ and a mean residence time of 18.2 h. CNCbl production began just after DMBI addition, reaching a volumetric production of 1.33 ± 0.26 mg/L during the first residence time. After 5 retention times, a stable OD_600_ value of around 24 was achieved, while glucose concentration remained stable at approximately 1.2–1.5 g/L. Propionic acid also presented a stable value of around 7.5 g/L, as expected much lower than the 20 g/L obtained in previous cultures and not in the inhibitory concentration range. These values were better than the expected ones, OD_600_ and propionic acid of 15 and 9 g/L respectively, so the continuous state not only allowed to control propionic acid at lower levels but also improved the carbon source usage towards biomass formation. Moreover, CNCbl volumetric production was stable from the first residence time at 1.33 ± 0.26 mg/L.

**Fig. 6:**
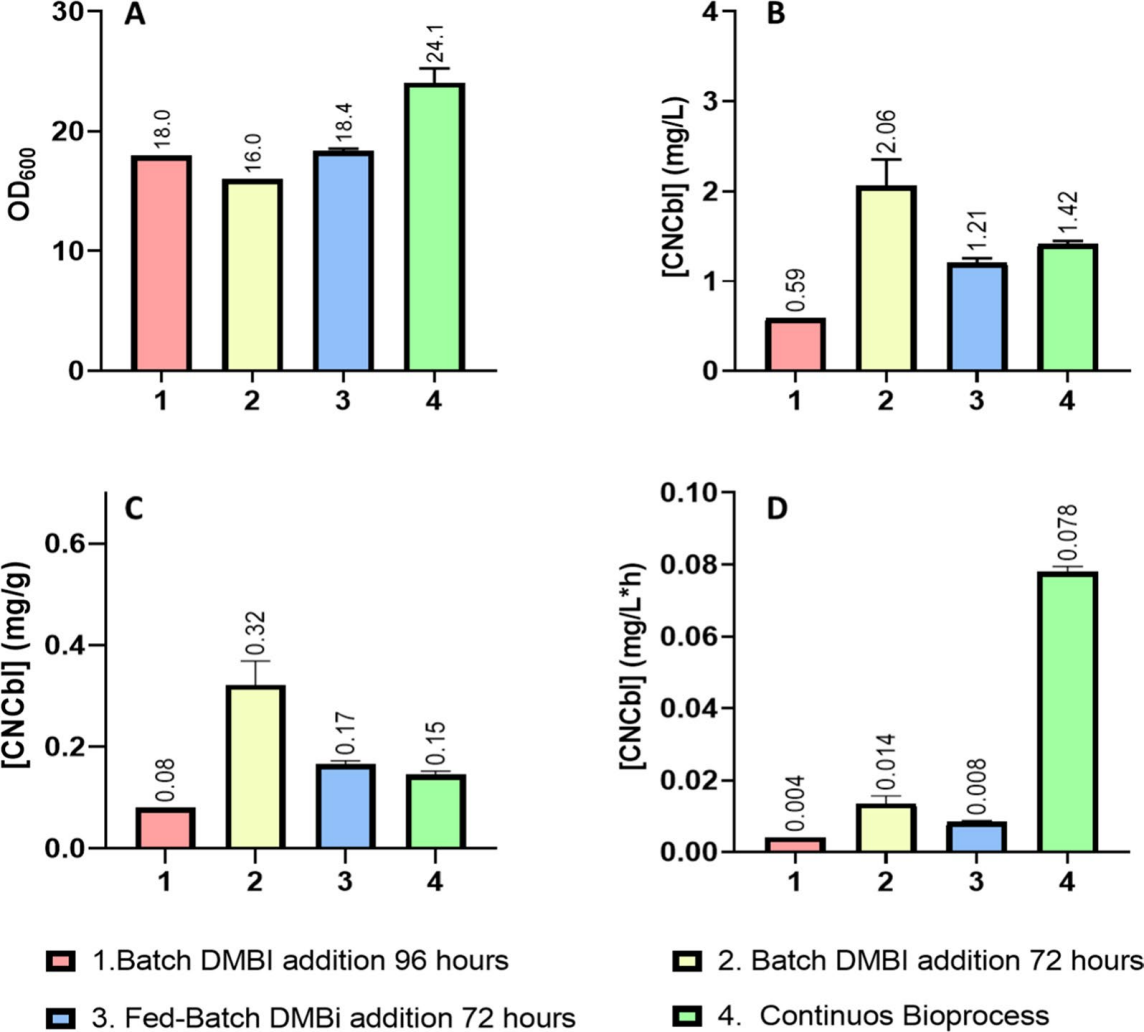
Comparison of several bioprocess parameters between the 4 scale-up strategies tested: batch with DMBI addition at 96 h, batch with DMBI addition at 72 h, fed-batch with DMBI addition at 72 h, and continuous bioprocess. **A** OD_600_. **B** Volumetric production. **C** Specific production. **D** Volumetric productivity. All data used to perform the calculations were extracted from the 144 h

Overall, comparing the two batch bioprocesses (with DMBI added at 96 and 72 h respectively), the fed-batch process and the newly developed continuous bioprocess at 144 h (Fig. 6), volumetric and specific production values showed values in the same range independently of the culture strategy. However, the volumetric productivities in the continuous process were 5.7-fold higher than the best batch process, 0.078 ± 0.001 vs 0.014 ± 0.002 mg CNCbl/(L·h). These results were possible due to the fact that the NBRC 12,391 strain can: (i) produce cobalamin in a single-phase anaerobic bioprocess, and (ii) its maximum volumetric production is achieved just 24 h after DMBI addition. However, the volumetric production is still lower than that obtained in static shake flask cultures, 2–4 mg CNCbl/L, giving space for further optimization.

In a review published recently by our group [38], the production of several wild-type *Propionibacterium* strains with different culture strategies is reported. The volumetric productivity achieved in this work is higher than most of the values obtained with wild-type strains [23, 38, 39]. Some other studies, like [40], reported higher volumetric productivities [0.32 mg CNCbl/(L·h)], most probably because a higher-producing strain was used.

## Conclusions

Despite the multiple limitations, mainly caused by its lower production and slower growth compared to aerobic strain producers, *P. freudenreichii* is still an interesting alternative for Cbl production, especially with the increasing demand for non-GMO natural products.

In this paper, suitable bioprocesses for CNCbl production with the wild-type strain NBRC 12391 were assessed in shake flasks and later scaled up to a 1L working volume bioreactor. It was determined that agitation during the early stages of the culture had a negative effect on final CNCbl productivity. Besides, DMBI was found to have a significant positive effect on final production. Specifically, our results showed that the time at which DMBI was added was important, with a slightly negative effect on cell growth when added a time 0 and maximum CNCbl volumetric productions just 24 h after DMBI addition, at 72 or 96 h depending on the culture strategy.

Batch with different addition times of DMBI and fed-batch processes were performed, but the results, especially the volumetric productivities, were hindered mainly by the lack of carbon source in the later stages of the culture and the inhibitory effect of propionic acid.

To increase CNCbl volumetric productivity of wild-type NBRC 12391, a single-phase continuous strategy was developed. The fact that the maximum CNCbl volumetric production with NBRC 12391 in our working conditions was achieved just after 24 h in anaerobic conditions, allowed us to achieve a 5.7-fold volumetric productivity increase following this strategy. Future work will address optimization regarding dilution rates and adjusting nutrient concentrations to achieve even higher volumetric productivities.

Overall, here we describe a new viable strategy for the development of future and sustainable strategies to produce CNCbl with wild-type GRAS* P. freudenreichii* strains without having to rely on more complicated setups like multiple-phase continuous bioprocesses or expanded bed adsorption bioreactors for propionic acid elimination.

## Materials and methods

### Microorganism and medium

Cultures were performed with *P. freudenreichii* NBRC 12391 acquired from Japan’s National Biological Resource Center (NBRC). This strain is the parental strain used by Kojima and co-workers in their Patent from 1985 [25], also named IFO 12391. Cells were stored in pre-culture media supplemented with 25% glycerol at − 80 °C and in agar-solid media.

All products described were purchased from Sigma-Aldrich unless stated otherwise.

The pre-culture media was prepared according to NBRC instructions. Its composition was 5 g/L glucose, 5 g/L yeast extract, 5 g/L tryptone and 1 g/L MgSO_4_. The composition of the agar-solid media was 5 g/L glucose, 5 g/L yeast extract, 5 g/L tryptone, 1 g/L MgSO_4_ and 15 g/L agar. Solid-media cultures were performed in a 2.5 L Oxoid Anaerojar (Thermo Fisher Scientific, Alcobendas, Spain) to ensure anaerobic conditions.

The composition of the culture medium used in shake flask and bioreactor cultures is an adaptation of the media described in Kojima et al. [25]. Its composition was 50 g/L glucose, 40 g/L corn steep liquor (CSL), 3 g/L NH_4_NO_3_, 1.5 g Na_2_HPO_4_, 0.4 g/L KH_2_PO_4_, 0.5 g/L MgSO_4_·7H_2_O, 5 mg/L MnSO_4_·4H_2_O, 10 mg/L FeSO_4_·7H_2_O, 10 mg/L ZnSO_4_·7H_2_O, 0.05 mg/L CuSO_4_·5H_2_O, 0.01 mg/L (NH_4_)_6_Mo_7_O_24_·4H_2_O, 15 mg/L Co(NO_3_)·6H_2_O and 5 mg/L Calcium pantothenate. Glucose and CSL concentrated stocks (250 g/L and 200 g/L stock solutions respectively) were autoclaved separately from the rest of the media components. To prevent precipitation of the CSL solid fraction, the pH of the CSL solution was adjusted to 8 before autoclaving and, after sterilization, centrifuged at 18,000*g* for 30 min to recover just the supernatant. All salts except NH_4_NO_3_, Na_2_HPO_4_, and KH_2_PO_4_ were prepared in a separated solution and sterilized by filtration with a 0.22 µm filter (Teknokroma, Sant Cugat del Valles, Spain).

For the development of a single-continuous bioprocess, the culture media composition was the same as previously described apart from glucose and CSL concentration which were set at 25 g/L and 20 g/L respectively.

### Shake flasks cultures

All shake flasks cultures were performed in 250 mL shake flasks filled with 200 mL of media at 30 °C with or without agitation depending on the study. The ratio between the volume of culture and the maximum volume of the shake flask was fixed at 80% to reduce the oxygenation capacity of the system as anaerobic and microaerobic conditions were desired.

An aliquot of the cryopreserved cells was grown in 10 mL of pre-culture media for 24 h. These cultures were used to inoculate another 10 mL of culture media for 48 h. Finally, these cultures were used to inoculate the main cultures used in the studies. pH was measured and adjusted daily with the addition of a 2 M NaOH solution. All cultures were performed, at least, in triplicate.

The effect of the agitation during the early stages of the culture was studied in two separate conditions: a static condition where cultures were kept in a stove (J.P. Selecta, Abrera,Spain) at 30 °C and an agitated culture performed at 150 rpm and 30 °C in a INFORS HT incubator (Biogen, Madrid, Spain). After 96 h, both conditions were supplemented with 100 µM DMBI and agitated at 150 rpm in the INFORS HT Incubator.

To study the effect of the addition of different cobalamin precursors, several cultures were performed with the addition of 100 µM Riboflavin (RF), 100 µM Nicotinamide (NAM), 100 µM DMBI or a combination of RF and NAM (50 µM each) at 0 and 96 h. All stock solutions for the different precursors were prepared with ultrapure water and filter sterilized except DMBI solution which was prepared in pure ethanol. The effect of DMBI addition on cell growth and production was further studied with the addition of 100 µM DMBI at 0, 48, 72 and 96 h. In all cases, after the addition of the precursors, cultures were kept under gently agitated conditions.

The viability of a glucose feeding strategy was firstly studied in shake flasks by the addition of a 250 g/L Glucose solution at 96 h to increase the remaining glucose concentration by 15 g/L. The inhibitory effect of propionic acid and the possibility of media regeneration was studied by replacing the media in the culture at 96 h by centrifugation at 3.900 g for 30 min and resuspending the cell pellets in 200 mL of fresh media.

Finally, the capability of NBRC 12391 to produce CNCbl in anaerobic conditions, without a microaeration phase, was performed in a stove without any agitation and using tight-closed lids during the whole process.

### Bioreactor cultures

All bioreactor cultures were performed using a Biostat A bioreactor (Sartorius Spain, Alcobendas, Spain) with a working final volume of 1 L. To ensure anaerobic conditions during the early stages of the culture, 0.150 vvm of N_2_ were constantly pumped into the bioreactor vessel through a 0.2 µm filter (Whatman, Maidstone, UK) and injected from the sparger. The agitation was maintained constant at 150 rpm. Temperature, pH and dissolved oxygen (pO_2_) were measured online by Hamilton probes. pH was adjusted constantly at 7.0 ± 0.05 with a 2 M NaOH solution. Temperature was constantly controlled at 30 °C.

In batch cultures, 1 L of fermentation media was inoculated with 10 mL of inoculum (1% v/v) to obtain an initial OD_600_ value of approximately 0.3. 100 µM of DMBI were added at 72 or 96 h depending on the culture and N_2_ addition was stopped to promote microaeration when desired. Air was not pumped during the cultures at any time.

In fed-batch processes, batch phase had 80% of the final volume and it was performed as previously described elsewhere. The fed phase began at 72 h with the addition of a concentrated glucose solution (250 g/L) at a constant rate of 6.66 mL/h. 100 µM of DMBI was also added to the culture at this point to promote Cbl production. A total amount of 200 mL of concentrated glucose solution was added to the culture for 30 h. After that time, no more glucose was added to the media and the culture was extended until 144 h.

Finally, for the continuous cultures, the batch phase was started as described before and from 48 h onwards fresh media with 100 µM DMBI were constantly added to the bioreactor vessel at 50 mL/h. Culture broth was constantly extracted from the bioreactor vessel by a level probe connected to a peristaltic bomb to ensure a constant working volume of 1.0 L.

### Analytical methods

Optic density at 600 nm (OD_600_) was determined with a JENWAY6305 spectrophotometer (Cole-Palmer, Staffordshire, UK).

Dry cell weight was calculated as follows. Periodically, 1 mL sample were centrifuged at 3900*g* for 15 min in triplicates in previously dried and weighed 15 mL falcon tubes. After centrifugation, the pellets were dried in a 100 °C oven until stable weight, cold down in a desiccator for 30 min and weighed again. The weight of the falcon tubes was taken into consideration for this calculation. After a series of cell dry weigh calculations in several cultures according to the method just described, it was established that one unit of OD_600_ corresponds to 0.405 g/L ± 0.02 of NBRC 12391 DCW (see Additional file 1 for more detailed information).

Glucose consumption and metabolite production, mainly acetate and propionic acids, were determined from supernatant samples. Briefly, at each sampling time, 1.0 mL of sample was centrifuged at 10,000*g* for 10 min and supernatants were separated, filtrated through 0.45 µm PVDF filters (Teknokroma, Sant Cugat del Valles, Spain) and stored at − 20 °C until its analysis. The metabolite analysis was performed by HPLC 1100 (Agilent, Santa Clara, USA) using an ICSep ICE-Coregel 87H3 column (Concise, San Jose, USA). A 0.05 M H_2_SO_4_ solution was used as a mobile phase at 0.5 mL/min. Organic acids were identified at different retention times with UV detection at 210 nm wavelength and glucose with the Refractive index detector. Acetic acid, propionic acid and glucose were quantified with 5-point calibration curve. Glucose was also measured during the cultures with a Y15 Biochemical analyzer (Biosystems, Barcelona, Spain).

Extraction and quantification of CNCbl were adapted from Wang et al. [29]. Briefly, cell pellets obtained from the centrifugation (10 min, 6500*g*), were resuspended in ultrapure water and heated at 90 °C for 20 min. After, they were cold down on ice and centrifuged at 3900*g* for 45 min. Supernatants were recovered and treated with a 0.1% (v/w) NaCN solution for 30 min at room temperature with periodical mixing to transform all possible cobalamin forms into CNCbl. NaCN was added in excess and should be enough to transform up to 200 mg/L of cobalamin. After filtration with 0.45 µm PVDF filters (Teknokroma, Sant Cugat del Valles, Spain), samples were analyzed by HPLC 1100 (Agilent, Santa Clara, USA) using a Waters Nova-Pak C18 4 µm 3.9X150 mm column. Mobile phase was composed of 70% Na_2_HPO_4_ anhydrous (10 g/L, pH at 3.5) and 30% methanol at a flow rate of 0.3 mL/min. CNCbl was detected by UV detector at 361 nm. Five CNCbl standards at different concentrations were injected in each quantification run to obtain the calibration curve.

## Supplementary Information

**Additional file 1: **Additional materials. **Table S1.** Evolution of DCW and OD600 in three independent cultures.

## Data Availability

The data that support the findings of this study are available from the corresponding authors upon reasonable request.
