# Supplementary material for: Developing a single-stage continuous process strategy for vitamin B12 production with Propionibacterium freudenreichii
Source: Microb Cell Fact. 2023 Feb 9;22:26. doi: 10.1186/s12934-023-02029-x (PMC9912679; doi:10.1186/s12934-023-02029-x)
Supplement: Supplementary file 1 — Additional file 1: Additional materials. Table S1. Evolution of DCW and OD600 in three independent cultures. [file 12934_2023_2029_MOESM1_ESM.pdf]

Additional file 1

# Developing a single-stage continuous process strategy for Vitamin $B_{12}$ production with *Propionibacterium freudenreichii subs shermanii*

Álvaro Calvillo[1], Teresa Pellicer[2], Marc Carnicer[1,*], Antoni Planas[1,*]

[1] Laboratory of Biochemistry, Institut Químic de Sarrià, University Ramon Llull, 08017 Barcelona, Spain; [2] HealthTech Bio Actives, 08029 Barcelona, Spain

*** Corresponding authors:** marc.carnicer@iqs.url.edu and antoni.planas@iqs.url.edu

## 1. Determination of Dry Cell Weight (DCW)/$OD_{600}$ ratio

In order to determine the DCW/$OD_{600}$ ratio for *P. freudenreichii* three independent cultures were performed where DCW and $OD_{600}$ were evaluated at different culture times (See Table S1).

*Table S1: Evolution of DCW and $OD_{600}$ in three independent cultures.*

| Time (h) | Culture 1 | | Culture 2 | | Culture 3 | |
|---|---|---|---|---|---|---|
| | $OD_{600}$ | DCW (g/L) | $OD_{600}$ | DCW (g/L) | $OD_{600}$ | DCW (g/L) |
| 0 | 0.05 | - | 0.05 | - | 0.05 | - |
| 24 | 1.85 | 0.78 | 1.75 | 0.71 | 1.71 | 0.75 |
| 48 | 10.0 | 3.98 | 10.3 | 4.08 | 10.0 | 4.0 |
| 72 | 22.0 | 9.07 | 21.5 | 9.0 | 20.9 | 8.1 |
| 96 | 29.0 | 11.2 | 29.5 | 11.7 | 29.0 | 11.9 |

The mean DCW/$OD_{600}$ ratio for each culture was 0.404, 0.404 and 0.408 respectively for cultures 1, 2 and 3. The established value for DCW/$OD_{600}$ ratio was the mean of each culture value, 0.405 ± 0.02.
